# Selenium Protects ARPE-19 and ACBRI 181 Cells against High Glucose-Induced Oxidative Stress

**DOI:** 10.3390/molecules28165961

**Published:** 2023-08-09

**Authors:** Handan Bardak, Abdülhadi Cihangir Uğuz, Yavuz Bardak, Javier Rocha-Pimienta, Jonathan Delgado-Adámez, Javier Espino

**Affiliations:** 1 Department of Ophthalmology, Asya Hospital, 34100 Istanbul, Turkey; handanbardak@yahoo.com.tr; 2 Department of Biophysics, Faculty of Medicine, Karamanoğlu Mehmetbey University, 70100 Karaman, Turkey; 3 Worldeye Hospital, 34337 Istanbul, Turkey; yavuzbardak@hotmail.com; 4 Technological Agri-Food Institute (CICYTEX-INTAEX), Junta of Extremadura, Avda. Adolfo Suárez s/n, 06007 Badajoz, Spain; javier.rocha@juntaex.es (J.R.-P.); jonathan.delgado@juntaex.es (J.D.-A.); 5 Department of Physiology, Faculty of Science, University of Extremadura, Avda. de Elvas, s/n, 06006 Badajoz, Spain

**Keywords:** ARPE-19 cells, high glucose, oxidative stress, selenium

## Abstract

Diabetic retinopathy (DR), a complication of diabetes mellitus (DM), can cause severe visual loss. The retinal pigment epithelium (RPE) plays a crucial role in retinal physiology but is vulnerable to oxidative damage. We investigated the protective effects of selenium (Se) on retinal pigment epithelium (ARPE-19) and primary human retinal microvascular endothelial (ACBRI 181) cells against high glucose (HG)-induced oxidative stress and apoptotic cascade. To achieve this objective, we utilized varying concentrations of D-glucose (ranging from 5 to 80 mM) to induce the HG model. HG-induced oxidative stress in ARPE-19 and ACBRI 181 cells and the apoptotic cascade were evaluated by determining Ca^2+^ overload, mitochondrial membrane depolarization, caspase-3/-9 activation, intracellular reactive oxygen species (ROS), lipid peroxidation (LP), glutathione (GSH), glutathione peroxidase (GSH-Px), vascular endothelial growth factor (VEGF) and apoptosis levels. A cell viability assay utilizing MTT was conducted to ascertain the optimal concentration of Se to be employed. The quantification of MTT, ROS, VEGF levels, and caspase-3 and -9 activation was accomplished using a plate reader. To quantitatively assess LP and GSH levels, GSH-Px activities were utilized by spectrophotometer and apoptosis, mitochondrial membrane depolarization, and the release of Ca^2+^ from intracellular stores were evaluated by spectrofluorometer. Our investigation revealed a significant augmentation in oxidative stress induced by HG, leading to cellular damage through modulation of mitochondrial membrane potential, ROS levels, and intracellular Ca^2+^ release. Incubation with Se resulted in a notable reduction in ROS production induced by HG, as well as a reduction in apoptosis and the activation of caspase-3 and -9. Additionally, Se incubation led to decreased levels of VEGF and LP while concurrently increasing levels of GSH and GSH-Px. The findings from this study strongly suggest that Se exerts a protective effect on ARPE-19 and ACBRI 181 cells against HG-induced oxidative stress and apoptosis. This protective mechanism is partially mediated through the intracellular Ca^2+^ signaling pathway.

## 1. Introduction

Diabetic retinopathy (DR), a complication of diabetes mellitus (DM), can cause severe visual loss [1]. DR arises from dysfunction within the retinal neurovascular unit, which encompasses cellular loss, the production of inflammatory factors, neovascularization, and impairment of the blood-retina barrier (BRB). The retinal pigment epithelium (RPE) plays a crucial role in retinal physiology but is vulnerable to oxidative damage [2,3]. Within the retina, the RPE cells form an integral component of the outer BRB. In DR, high glucose (HG) levels have been shown to induce reactive oxygen species (ROS) and cell apoptosis [4,5]. Apoptosis can be triggered by increased intracellular calcium ([Ca^2+^]_i_) and ROS levels. Therefore, transient receptor potential (TRP) channels responsible for Ca^2+^ movements between extracellular and cytosolic media have important functions in RPE cell physiology [6,7,8]. Caspase-3 and -9 proteins have been found to be effective in the regulation of apoptosis [9]. Vascular endothelial growth factor (VEGF) secreted by RPE cells is needed for the physiological function of the choriocapillaris. VEGF levels have been shown to increase in DR patients [10].

Selenium (Se), an essential dietary trace element and useful antioxidant, functions as a co-factor of antioxidant enzymes such as glutathione peroxidase (GSH-Px) [11]. Ion channels are the gatekeepers of the cell membrane, allowing ions movement through the cell membrane. Several types of cation channel components have been recognized as target molecules of different compounds that accumulate during hyperglycemia. For instance, high glucose levels, a hallmark of diabetes, can activate TRPC channels, leading to increased [Ca^2+^]_i_ and subsequent cellular dysfunction. Thus, the Ca^2+^ influx can trigger downstream signaling events that promote oxidative stress, apoptosis, and abnormal angiogenesis. Diabetes-related reactive metabolites, including reactive oxygen and nitrogen species, also affect the ion channel activity. TRP channels, particularly canonical type TRP (TRPC) channels, are sensitive to these molecules in the retina [12]. Former studies clearly demonstrated the altered activity of the TRPC5 channel after thioredoxin [13] or oxidized glutathione application [14]. The development of DR is triggered by the accumulation of reactive metabolites, including ROS. These metabolites have been shown to influence the functioning of cation channels belonging to the TRPC family, which were found as redox-sensitive channels [15].

The development of long-term diabetic complications associated with hyperglycemia is driven by the accumulation of reactive metabolites. Recent studies have revealed that these metabolites have an impact on cation channels, which serve as targets in signaling pathways implicated in the pathogenesis of the disease. However, no detailed studies on molecular mechanisms effective in HG-induced TRP channel activation and oxidative stress in RPE and ACBRI 181 cells are available. By using different HG models to induce oxidative stress in ARPE-19 and ACBRI 181 cells, the current study demonstrated for the first time the protective role of Se and its regulatory function on TRPC channels. To understand this interaction between the HG model and the TRP channel, the protective actions of Se treatment on apoptosis, caspase-3/-9 activation, oxidative stress levels, VEGF expression, and [Ca^2+^]_i_ release in ARPE-19 cells were investigated.

## 2. Results

### 2.1. Toxic and Therapeutic Doses of Se on Cell Viability

**Figure 1:**
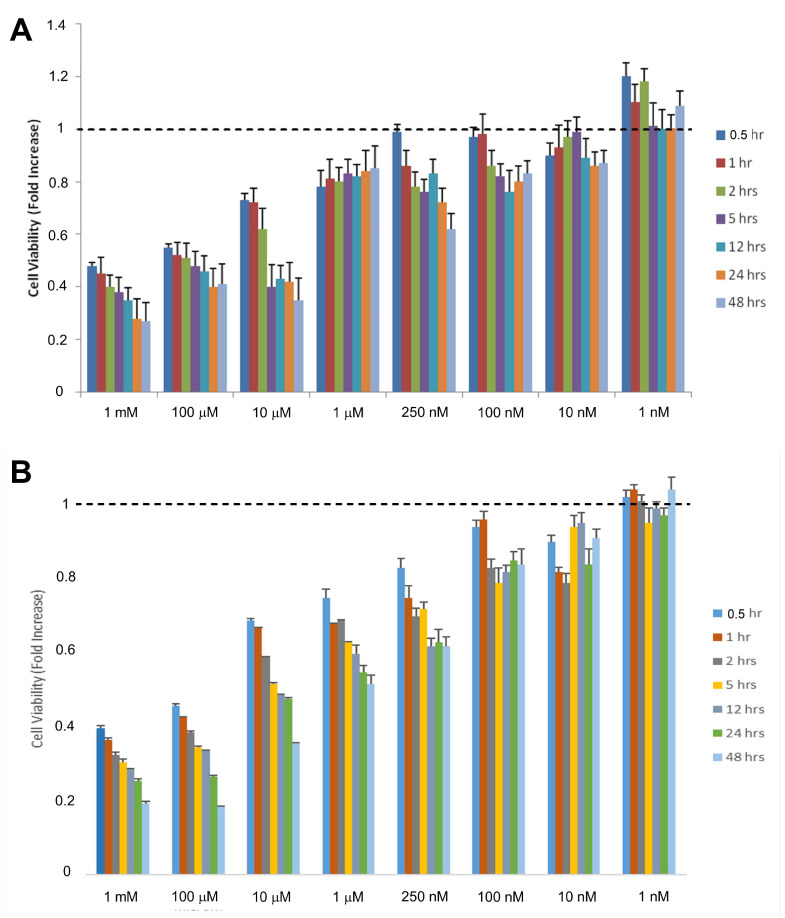
Effect of selenium (Se) on cell viability in ARPE-19 (**A**) and ACBRI 181 (**B**) cells (*n* = 5). Cells were incubated with increasing concentrations of Se (1 nM–1 mM) for various time points (0.5–48 h).

Eight different doses of Se (0.001–1 µM) were investigated by the cell viability test of ARPE-19 (Figure 1A) and ACBRI 181 (Figure 1B) cells. Cell viability tests were performed at seven different time points (0.5, 1, 2, 5, 12, 24, 48 h). There was statistical significance in eight doses of Se. Toxic effects of Se started at a 1 mM dose (*p* < 0.05). The total percentage of live cells was lower than 50% in 1 mM of Se. We first chose the incubation for the dose and duration close to 50% viability when evaluating cell viability percentages as 250 nM and 48 h. Cell viability significantly decreased (*p* < 0.001) in 1 mM dose of Se at 1 h. We determined a statistically significant (*p* < 0.01) decrease (>50%) in cell viability at 10 µM Se during 2 h of incubation. The toxic effects started with 10 µM Se at 5 h. In the current study, around 60% cell viability was observed when cells were challenged with 250 nM Se for 48 h, showing similarity to previous reports [16,17]. Similarly, 250 nM Se for 24 h incubation was performed for ACBRI 181 cells.

### 2.2. Effects of Se and HG on Intracellular Ca^2+^ Concentrations

**Figure 2:**
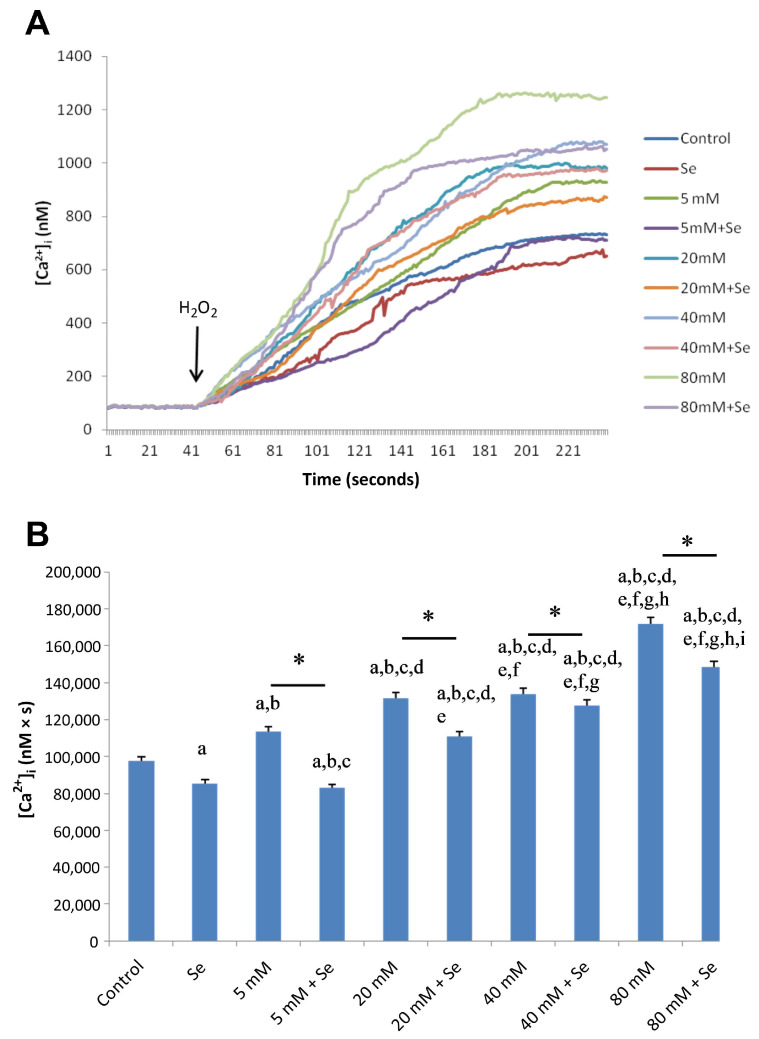
Calcium release from ARPE-19 cells exposed to selenium (Se) and different concentrations of D-glucose (5–80 mM) and their combinations (**A**). Fura-2, AM-loaded ARPE-19 cells were incubated for 45 min in a shaking water bath. Subsequently, cells were exposed to 100 µM H_2_O_2_ to induce stimulation. Time course chart recordings were taken to visualize the transient [Ca^2+^]_i_ levels in ARPE-19 cells. (**B**) Bar charts present the mean ± standard deviation data representing [Ca^2+^]_i_ concentration in H_2_O_2_-stimulated ARPE-19 cells (*n* = 6 for each group). A single asterisk indicates significant differences between the two groups (*p* < 0.001). ^a^ *p* < 0.001 vs. control, ^b^ *p* < 0.001 vs. Se, ^c^ *p* < 0.001 vs. 5 mM D-glucose, ^d^ *p* < 0.001 vs. 5 mM D-glucose + Se, ^e^ *p* < 0.001 vs. 20 mM D-glucose, ^f^ *p* < 0.001 vs. 20 mM D-glucose + Se, ^g^ *p* < 0.001 vs. 40 mM D-glucose, ^h^ *p* < 0.001 vs. 40 mM D-glucose + Se, ^i^ *p* < 0.001 vs. 80 mM D-glucose.

**Figure 3:**
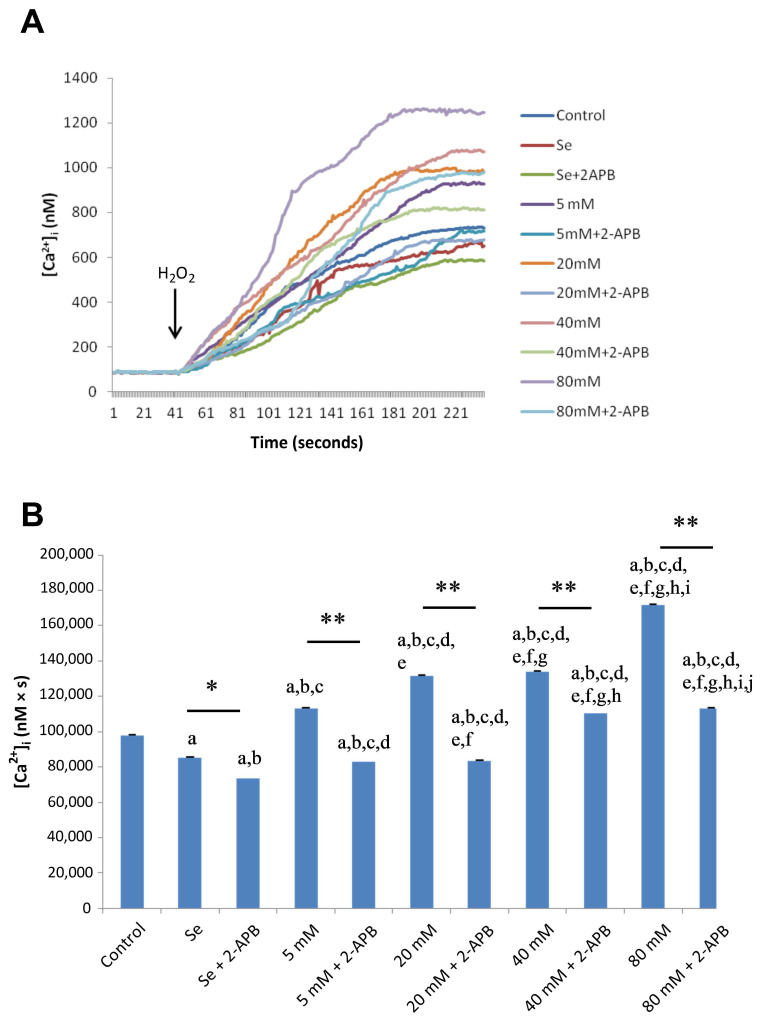
Calcium release from ARPE-19 cells exposed to selenium (Se), different concentrations of D-Glucose (5–80 mM), and their combinations after 2-APB incubation calcium release from ARPE-19 cells (**A**). Fura-2, AM-loaded ARPE-19 cells were incubated for 45 min in a shaking water bath. Subsequently, cells were exposed to 100 µM H_2_O_2_ to induce stimulation. Time course chart recordings were taken to visualize the transient changes in [Ca^2+^]_i_ levels in ARPE-19 cells. (**B**) Bar charts present the mean ± standard deviation data representing [Ca^2+^]_i_ concentration in H_2_O_2_-stimulated ARPE-19 cells (*n* = 6 for each group). A single asterisk (*) indicates significant differences between two groups (*p* < 0.05), and double asterisks (**) indicate significant differences between two groups (*p* < 0.001). ^a^ *p* < 0.001 vs. control, ^b^ *p* < 0.001 vs. Se, ^c^ *p* < 0.001 vs. Se + 2-APB, ^d^ *p* < 0.001 vs. 5 mM D-glucose, ^e^ *p* < 0.001 vs. 5 mM D-glucose + 2-APB, ^f^ *p* < 0.001 vs. 20 mM D-glucose, ^g^ *p* < 0.001 vs. 20 mM D-glucose + 2-APB, ^h^ *p* < 0.001 vs. 40 mM D-glucose, ^i^ *p* < 0.001 vs. 40 mM D-glucose + 2-APB, ^j^ *p* < 0.001 vs. 80 mM D-glucose.

In the experimental HG group, the mean cytosolic Ca^2+^ concentration significantly increased in a dose-dependent manner in ARPE-19 cells, a trend that was significantly counteracted by Se (Figure 2A,B). However, pre-incubation with 2-aminoethoxydiphenyl borate (2-APB, 75 µM), a specific TRPC1 channel blocker, significantly decreased HG-mediated Ca^2+^ entry via the TRPC1 cation channel (Figure 3A,B). In all cases, Ca^2+^ values were found to be significantly lower in the control group and Se groups compared to HG groups (*p* < 0.001).

**Figure 4:**
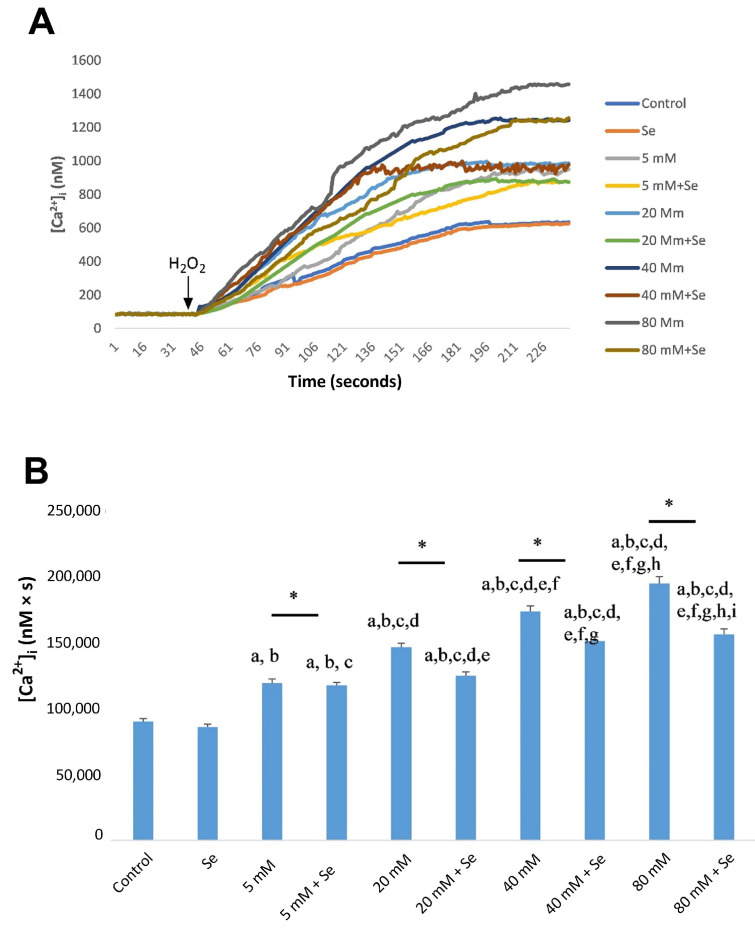
Calcium release from ACBRI 181 cells exposed to selenium (Se), different concentrations of D-glucose (5–80 mM), and their combinations (**A**). Fura-2, AM-loaded ACBRI 181 cells were incubated for 45 min in a shaking water bath. Subsequently, cells were exposed to 100 µM H_2_O_2_ to induce stimulation. Time course chart recordings were taken to visualize the transient changes in [Ca^2+^]_i_ levels in ACBRI 181 cells. (**B**) Bar charts present the mean ± standard deviation data representing [Ca^2+^]_i_ concentration in H_2_O_2_-stimulated ARPE-19 cells (*n* = 6 for each group). A single asterisk indicates significant differences between the two groups (*p* < 0.001). ^a^ *p* < 0.001 vs. control, ^b^ *p* < 0.001 vs. Se, ^c^ *p* < 0.001 vs. 5 mM D-glucose, ^d^ *p* < 0.001 vs. 5 mM D-glucose + Se, ^e^ *p* < 0.001 vs. 20 mM D-glucose, ^f^ *p* < 0.001 vs. 20 mM D-glucose + Se, ^g^ *p* < 0.001 vs. 40 mM D-glucose, ^h^ *p* < 0.001 vs. 40 mM D-glucose + Se, ^i^ *p* < 0.001 vs. 80 mM D-glucose.

**Figure 5:**
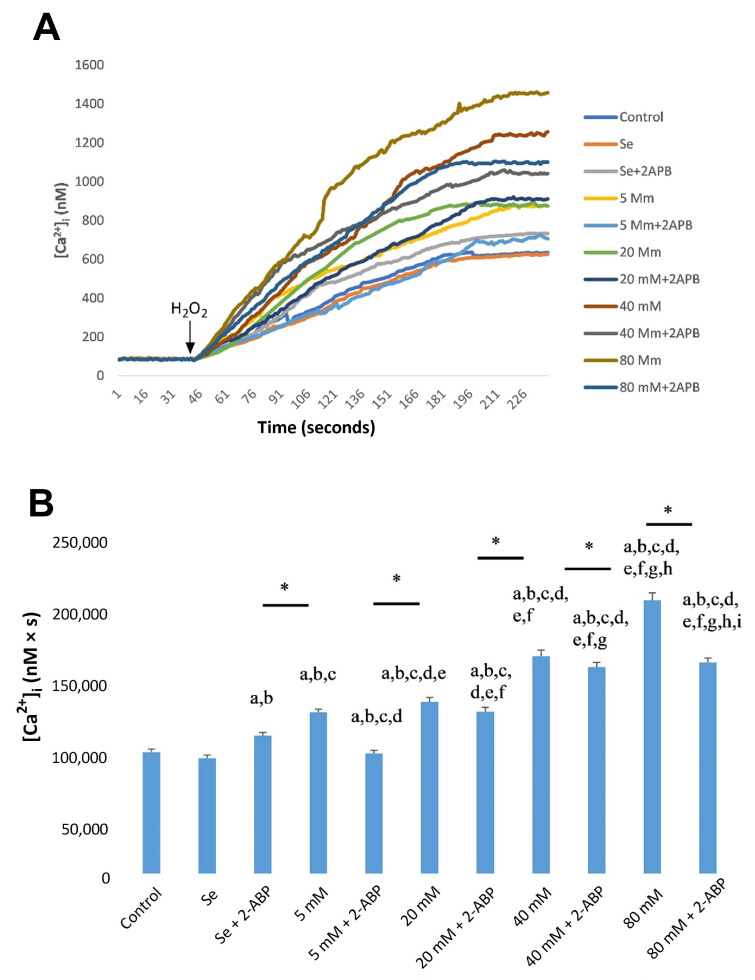
Calcium release from ACBRI 181 cells exposed to selenium (Se), different concentrations of D-Glucose (5–80 mM), and their combinations after 2-APB incubation calcium release from ACBRI 181 cells (**A**). Fura-2, AM-loaded ACBRI 181 cells were incubated for 45 min in a shaking water bath. Subsequently, cells were exposed to 100 µM H_2_O_2_ to induce stimulation. Time course chart recordings were taken to visualize the transient changes in [Ca^2+^]_i_ levels in ACBRI 181 cells. (**B**) Bar charts present the mean ± standard deviation data representing [Ca^2+^]_i_ concentration in H_2_O_2_-stimulated ARPE-19 cells (*n* = 6 for each group). A single asterisk (*) indicates significant differences between two groups (*p* < 0.05). ^a^ *p* < 0.001 vs. control, ^b^ *p* < 0.001 vs. Se, ^c^ *p* < 0.001 vs. Se + 2-APB, ^d^ *p* < 0.001 vs. 5 mM D-glucose, ^e^ *p* < 0.001 vs. 5 mM D-glucose + 2-APB, ^f^ *p* < 0.001 vs. 20 mM D-glucose, ^g^ *p* < 0.001 vs. 20 mM D-glucose + 2-APB, ^h^ *p* < 0.001 vs. 40 mM D-glucose, ^i^ *p* < 0.001 vs. 40 mM D-glucose + 2-APB.

As for ACBRI 181 cells, in the experimental HG group, the mean cytosolic Ca^2+^ concentration was significantly increased in a dose-dependent way, which was significantly counteracted by Se (Figure 4A,B). However, pre-incubation with 2-APB significantly decreased HG-mediated Ca^2+^ entry via the TRPC1 cation channel (Figure 5A,B). In all cases, Ca^2+^ values were found to be significantly lower in the control group and Se groups compared to HG groups (*p* < 0.001).

### 2.3. Effects of Se and HG on Intracellular ROS Production

**Figure 6:**
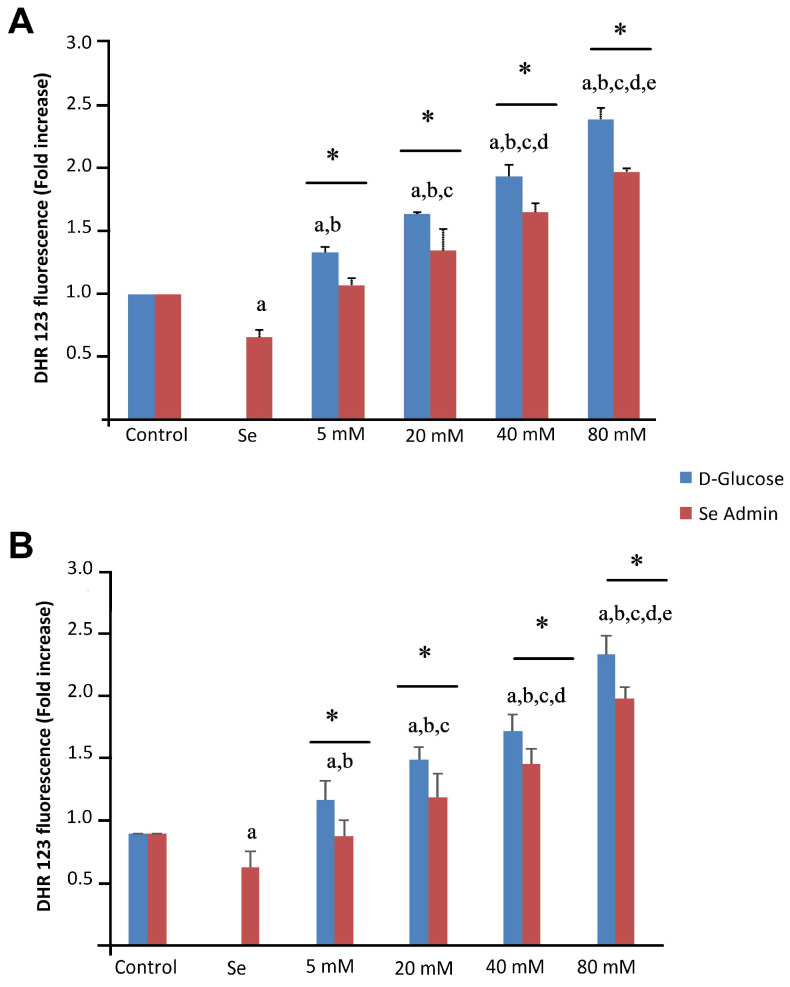
Effects of selenium (Se) and high-glucose (HG) on intracellular ROS production of ARPE-19 (**A**) and ACBRI 181 (**B**) cells. Red bars indicate Se administered groups, and blue bars indicate only D-glucose administered groups. A single asterisk indicates significant differences between each group (*p* < 0.05). ^a^ *p* < 0.001 vs. control, ^b^ *p* < 0.001 vs. Se, ^c^ *p* < 0.001 vs. 5 mM D-glucose, ^d^ *p* < 0.001 vs. 20 mM D-glucose, ^e^ *p* < 0.001 vs. 40 mM D-glucose.

Figure 6 illustrates the mean levels of cytosolic ROS production in both ARPE-19 (Figure 6A) and ACBRI 181 (Figure 6B) cells. The levels of intracellular ROS production were significantly higher (*p* < 0.001) in the HG group than in the control and Se groups.

### 2.4. Effects of Se and HG on Mitochondrial Membrane Depolarization Levels

**Figure 7:**
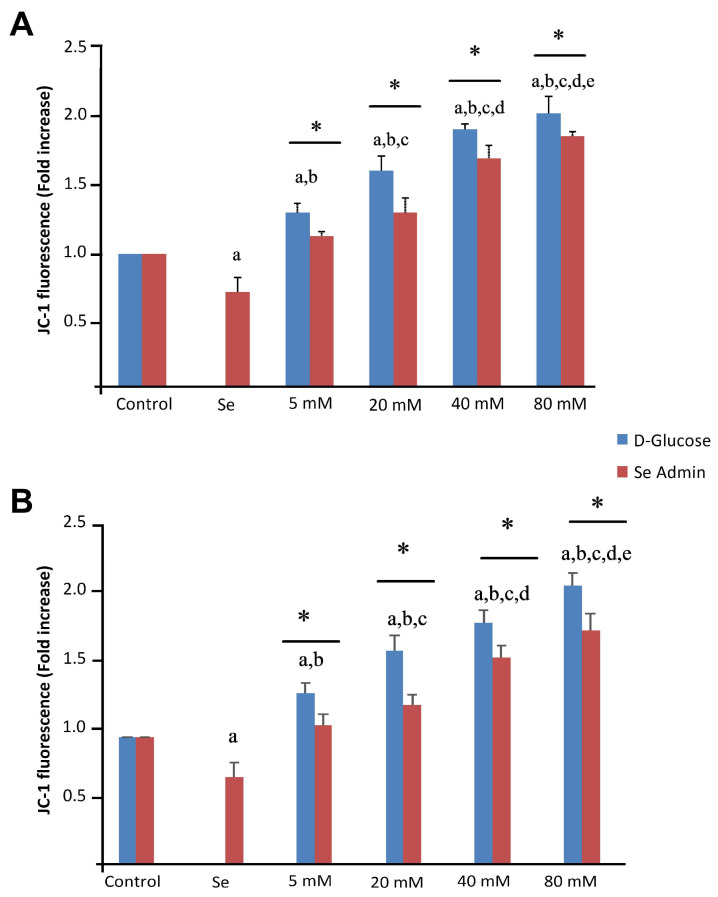
Effects of selenium (Se) and high-glucose (HG) on mitochondrial membrane depolarization of ARPE-19 (**A**) and ACBRI 181 (**B**) cells. Red bars indicate Se administered groups, and blue bars indicate only D-glucose administered groups. A single asterisk indicates statistically significant differences between each group (*p* < 0.05). ^a^ *p* < 0.001 vs. control, ^b^ *p* < 0.001 vs. Se, ^c^ *p* < 0.001 vs. 5 mM D-glucose, ^d^ *p* < 0.001 vs. 20 mM D-glucose, ^e^ *p* < 0.001 vs. 40 mM D-glucose.

The JC-1 levels in ARPE-19 and ACBRI 181 cells were significantly (*p* < 0.001) higher in HG group cells than in the control group (Figure 7A,B). Se incubation significantly decreased mitochondrial membrane depolarization levels (*p* < 0.001; Figure 7).

### 2.5. Effects of Se and HG on Apoptosis and Caspase-3/-9 Activation

**Figure 8:**
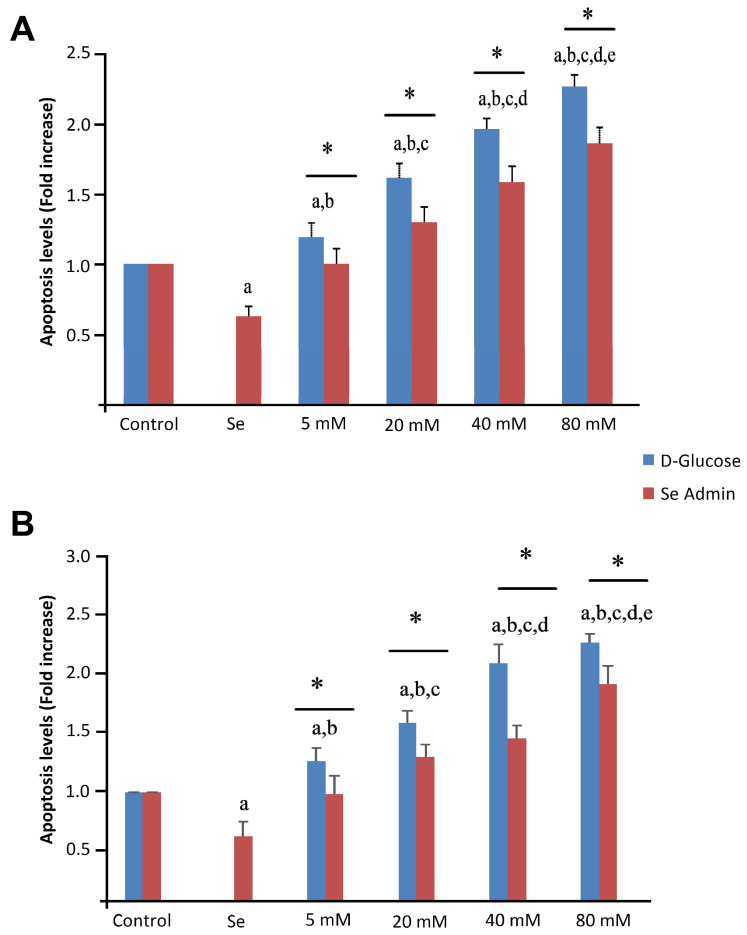
Effects of selenium (Se) and high-glucose (HG) on apoptosis of ARPE-19 (**A**) and ACBRI 181 (**B**) cells. Red bars indicate Se administered groups, and blue bars indicate only D-glucose administered groups. A single asterisk indicates statistically significant differences between each group (*p* < 0.05). ^a^ *p* < 0.001 vs. control, ^b^ *p* < 0.001 vs. Se, ^c^ *p* < 0.001 vs. 5 mM D-glucose, ^d^ *p* < 0.001 vs. 20 mM D-glucose, ^e^ *p* < 0.001 vs. 40 mM D-glucose.

**Figure 9:**
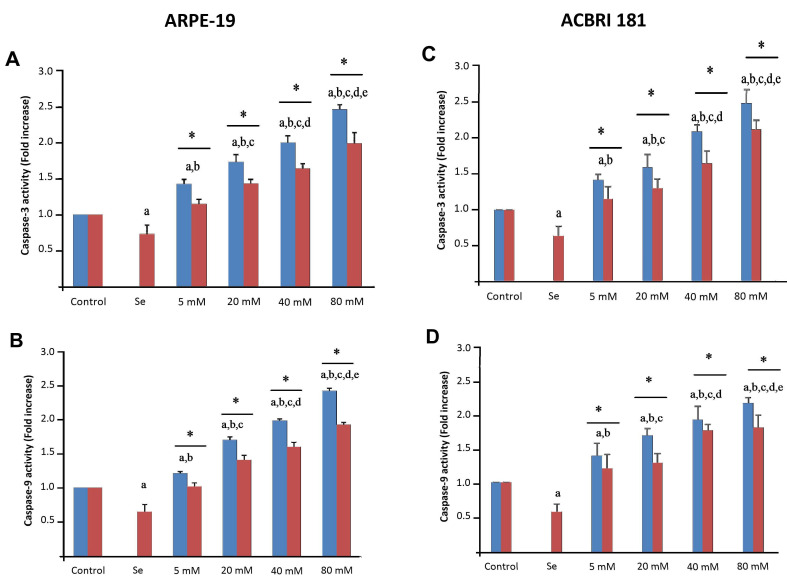
Effects of selenium (Se) and high-glucose (HG) on caspase-3 (**A**,**C**) and -9 (**B**,**D**) activation in ARPE-19 (left panels) and ACBRI 181 (right panels) cells. Red bars indicate Se administered groups, and blue bars indicate only D-glucose administered groups. A single asterisk indicates statistically significant differences between each group (*p* < 0.05). ^a^ *p* < 0.001 vs. control, ^b^ *p* < 0.001 vs. Se, ^c^ *p* < 0.001 vs. 5 mM D-glucose, ^d^ *p* < 0.001 vs. 20 mM D-glucose, ^e^ *p* < 0.001 vs. 40 m M D-glucose.

Caspase-3 and -9 activation and apoptosis induction in ARPE-19 and ACBRI 181 cells untreated (control) and treated with Se, different dosages of D-glucose and their combinations are shown in Figure 8 and Figure 9, respectively. The proportion of apoptotic cells (*p* < 0.001) as well as caspase-3 (*p* < 0.001) and caspase-9 (*p* < 0.001) activities were found to be significantly higher in the HG groups than in the control and Se groups (Figure 8 and Figure 9). Se alone, or combined administration of Se and D-glucose, significantly decreased apoptosis and caspase-3 and -9 activation (Figure 8 and Figure 9). Furthermore, statistically significant differences were noticed between different groups (indicated with asterisks).

### 2.6. Effects of Se and HG on LP, GSH, GSH-Px, and VEGF Levels

The mean LP levels of the ARPE-19 and ACBRI 181 cells are shown in Table 1 and Table 2, respectively. The LP levels (µmol/g protein) were significantly (*p* < 0.001) higher in the HG groups than in the control groups. Combination with Se significantly (*p* < 0.001) decreased LP levels. It is also shown that GSH-Px activities and GSH concentrations were significantly (*p* < 0.001) lower in the HG groups than in the controls. Nevertheless, Se administration significantly (*p* < 0.001) increased GSH and GSH-Px levels. Additionally, the HG model significantly increased (*p* < 0.001) VEGF levels; however, Se normalized HG-induced VEGF levels.

## 3. Discussion

Several possible molecular pathways of HG-induced oxidative stress in RPE cells in vitro were identified. Our results demonstrated that incubation with D-glucose triggers oxidative stress and promotes alterations in mitochondrial depolarization levels as well as [Ca^2+^]_i_ levels. HG model significantly reduced GSH-Px and GSH levels. GSH-Px plays an important role in converting hydrogen peroxide (H_2_O_2_) to a water molecule (H_2_O). H_2_O_2_ is also responsible for gating some of the Ca^2+^ channels [18]. Excessive ROS production, alterations in mitochondrial depolarization levels, TRP channel activation, and Ca^2+^ overload can be effective mechanisms in the apoptotic process. These mechanisms have been shown to be effective in HG-induced DR pathogenesis [19,20,21]. Also, HG causes apoptosis in cardiac, pancreatic beta and pancreatic islet cells [4,22]. Chen et al. reported that 40 mM D-glucose significantly induced apoptosis in ARPE-19 cells [23]. In the current study, caspase-3 and -9 levels showed a significant increase starting from 20 mM D-glucose. These findings were in agreement with the results of Chen et al. [23]. In the current study, Se incubation was demonstrated to significantly regulate Ca^2+^ release from intracellular stores and significantly decrease Ca^2+^-related apoptosis. This finding may help to understand better the role of TRPC channels in DR. As a result, the HG-induced ROS production showed a remarkable decrease by Se treatment as Ca^2+^ influx was inhibited. The extended duration of Ca^2+^ channel activation generates more transient Ca^2+^ signals that contribute to membrane ionic current alterations and intracellular Ca^2+^ homeostasis defects [24], which were similar to our findings. When the oxidative stress is not buffered by the mitochondria, the apoptotic processes are triggered [25,26]. One study showed that ROS production and stimulation of apoptotic caspase-3-and -9-dependent pathways get increased in the retina by amplified mitochondrial membrane depolarization [27]. We think that retinal oxidative damage results from a deficiency of the antioxidant defense system. Our results suggested that the antioxidant drug Se significantly decreased ROS generation and apoptosis via the regulation of mitochondrial functions in the RPE cells. One previous study documented that HG-induced oxidative stress increases VEGF levels in DR [28]. VEGF is a potent signaling protein that stimulates the growth of new blood vessels (angiogenesis) and increases vascular permeability. While these processes are essential for tissue repair and development, in the context of DR, excessive VEGF levels may contribute to the development of abnormal and leaky blood vessels in the retina, a condition known as neovascularization. Since VEGF plays a central role in the pathogenesis and progression of the eye’s condition, its importance in DR cannot be overstated. High glucose levels in the retina can lead to increased expression of VEGF, contributing to the development of DR. In DR, prolonged high blood sugar levels cause damage to the blood vessels, leading to retinal ischemia (insufficient blood supply) and hypoxia (lack of oxygen). In response to the hypoxic conditions, the body increases the production of VEGF. The study of VEGF levels in diabetic retinopathy using ARPE-19 cells is of great importance to understanding the underlying mechanisms of this sight-threatening condition and is achieved by mimicking the retinal pigment epithelial cells found in the human retina. VEGF levels play a role in promoting abnormal blood vessel growth and vascular leakage, which are hallmark features of diabetic retinopathy. Moreover, VEGF-induced vascular leakage results in the accumulation of fluid and proteins in the retinal tissue, causing macular edema, another vision-threatening complication of DR. This fluid accumulation disrupts the normal retinal architecture and impairs vision by distorting or blurring central vision. We observed that HG significantly increased VEGF levels in RPE cells in a dose-dependent manner. However, Se significantly decreased VEGF synthesis in RPE cells. ACBRI 181 cells showed similar results. This effect of Se can be explained by its antioxidant property and its effects on intracellular signaling pathways. Protein kinase C (PKC) was shown to reduce VEGF-induced vascular hyperpermeability in diabetic mice [29]. Similarly, Titchenell et al. also demonstrated that PKC downregulates VEGF-induced barrier damage [30]. PKC enzymes can be stimulated by intracellular signals such as elevated concentrations of Ca^2+^ or diacylglycerol [31]. These results indicate the efficacy of Se in various signal transduction cascades as an antioxidant. Moreover, Gonzalez et al. also determined the protective effect of Se administration in RPE cells, which was dependent on the production of new glutathione molecules and preventing GSH-Px from losing its activity [32].

Various retrospective studies investigating the correlation between plasma selenium and the risk of DR have produced conflicting findings. For instance, Kurt et al. [33] observed that patients with diabetic retinopathy had lower plasma selenium levels compared to the control group, whereas Wang et al. [34] found significantly higher plasma and vitreous selenium concentrations in DR patients compared to control patients. Additionally, in a cross-sectional study of the United States population, selenium was found to be positively associated with diabetes but inversely associated with all-cause mortality [35]. One possible explanation for these contradictory results could be the dual impact of selenium on insulin secretion and action. Optimal levels of selenium may enhance insulin sensitivity and glucose regulation, but excessive selenium could promote insulin resistance, leading to the development of T2D. Given these controversial findings, further research is necessary to comprehensively understand the relationship between plasma selenium and DR risk, as well as the ideal selenium levels for metabolic health in individuals with diabetes. As a precaution, readers and the general population should exercise caution when considering selenium supplements.

Furthermore, in this study, some important clues were determined that support the hypothesis that HG-induced oxidative stress leads to dysregulation of Ca^2+^ cascades, Ca^2+^ re-uptake into mitochondria, mitochondrial membrane depolarization, excessive free oxygen radical production, and apoptosis levels. Additionally, Se was shown to exert a protective effect against HG-induced oxidative stress in RPE cells.

## 4. Materials and Methods

### 4.1. Chemicals

D-glucose, potassium hydroxide (KOH), glutathione, sodium hydroxide (NaOH), sodium selenite, nitrobenzoic acid, triton X-100, thiobarbituric acid, 1,1,3,3-tetraethoxypropane, tris-hydroxymethyl-aminomethane, 5,5-dithiobis-2 cumene hydroperoxide, butylhydroxytoluol (BHT), and ethylene glycol-bis [2-aminoethyl-ether]-N, N, N, N-tetraacetic acid [EGTA]) were obtained from Sigma-Aldrich (St. Louis, MO, USA). Fura-2 acetoxymethyl (AM) ester was procured from Invitrogen (Carlsbad, CA, USA). N-hexane and ethyl alcohol were purchased from Merck (Darmstadt, Germany). All the reagents used were of analytical grade. Except for the phosphate buffers, all reagents were prepared daily and stored at 4 °C. Before the analysis commenced or reagent containers were refilled, the reagents were equilibrated at room temperature for thirty minutes. Dulbecco’s Modified Eagle’s Medium and Ham’s F-12 (DMEM/F-12) medium, fetal bovine serum (FBS), and a penicillin-streptomycin combination were obtained from Biochrom (Biochrom, Berlin, Germany). Biocolor (Belfast, Northern Ireland) supplied the APOPercentage^TM^ assay kit.

### 4.2. Cell Culture

The human RPE cell line ARPE-19 (ATCC, Manassas, VA, USA) was cultivated using a medium consisting of a 1:1 ratio of DMEM/F-12 medium, supplemented with 10% FBS and 1% penicillin-streptomycin combination, following the manufacturer’s standard protocol [36]. Primary retinal microvascular endothelial cells (ACBRI 181) (Cell Systems, Seattle, WA, USA) were cultured in M199 medium with 20% fetal bovine serum, 3 ng/mL FGF basic, 10 units/mL heparin, and 1% penicillin-streptomycin combination. The cell lines were routinely cultured at 37 °C in a humidified incubator with 5% CO_2_. Cells were used in passages four to twelve. To prepare the treatments, D-glucose and Se were dissolved in DMEM/F-12, resulting in final concentrations of 5, 20, 40, or 80 mM D-glucose and 250 nM Se. For the HG treatment, cells were seeded in a 48-well plate and cultured until reaching a higher cell confluence of 85%. Subsequently, the cells were treated with the above-mentioned D-glucose concentrations for a duration of 48 h. The control medium contained glucose concentrations four times lower than 5 mM.

### 4.3. Determination of Selenium Doses by Cell Viability (MTT Assay)

MTT assay was used for cell viability. ARPE-19 cells and ACBRI 181 cells were seeded in 25 cm^2^ flasks at a density of 2 × 10^6^/tube and subsequently exposed to several concentrations of Se (10 nM–1 mM) and at different incubation periods (1–48 h) at 37 °C. Following the treatments, the medium was removed from each tube, and MTT was introduced. The tubes were then incubated in a shaking water bath at 37 °C for 90 min. After incubation, the supernatant was discarded, and dimethyl sulfoxide (DMSO) was added to dissolve the formazan crystals. The treatments were performed in duplicate. Optical density measurements were obtained using an automatic microplate reader (Tecan Infinite M200 for ARPE-19 cells and SpectroStar Nano [BMG LabTech] (Ortenberg, Germany) for ACBRI 181 cells) at 490 and 650 nm (as reference wavelengths). The results were presented as the fold increase over the pretreatment level (experimental/control).

### 4.4. Determination of Calcium ([Ca^2+^]_i_) by Fluorescent Dye

According to the manufacturer’s protocol, cells were loaded with 4 µM Fura-2, AM [26]. Thereafter, they were washed and gently resuspended in Na-HEPES solution containing (in mM): NaCl, 140; KCl, 4.7; CaCl_2_, 1.2; MgCl_2_, 1.1; glucose, 10; and HEPES, 10 (pH 7.4). The groups were exposed to D-glucose for stimulating ([Ca^2+^]_i_) release. Fluorescence was recorded from 2 mL aliquots of magnetically stirred cellular suspension (2 × 10^6^ cells/mL) at 37 °C by using a spectrofluorometer (Cary Eclipse, Varian Inc., Sydney, Australia) with excitation wavelengths of 340 and 380 nm and emission at 505 nm. The changes in [Ca^2+^]_i_ were monitored by measuring the Fura-2, AM 340/380 nm fluorescence ratio and calibrated based on the method described by Grynkiewicz et al. [37]. The estimation of Ca^2+^ release was determined by calculating the integral of the rise in [Ca^2+^]_i_ for 200 s after the addition of H_2_O_2_ [38]. The measurement of Ca^2+^ release involves sampling every second (nM/s), following the previously established methodology [39].

### 4.5. Measurement of ROS-Sensitive Fluorescence

Determination of intracellular ROS production was carried out by loading cells with 2 µM dihydrorhodamine-123 (DHR-123) and measuring the resulting fluorescence intensity in an automatic microplate reader (Tecan Infinite M200 for ARPE-19 cells and SpectroStar Nano [BMC LabTech] for ACBRI 181 cells), as previously described [18]. Data were presented as the fold increase over the control level (untreated samples).

### 4.6. Measurement of Lipid Peroxidation (LP) Level

Determination of LP levels in the ARPE-19 and ACBRI 181 cell lines was carried out by measuring the thiobarbituric-acid reaction, as previously described [40]. Thiobarbituric acid reactive substances were measured by comparing their absorption to the standard curve of malondialdehyde equivalents produced through acid-catalyzed hydrolysis of 1,1,3,3-tetramethoxypropane.

### 4.7. Measurement of Reduced Glutathione (GSH), Glutathione Peroxidase (GSH-Px), and Protein Assay

The method described by Sedlak and Lindsay was used to ascertain the GSH content of the ARPE-19 and ACBRI 181 cells, measured at 412 nm [41]. According to the Lawrence and Burk method, the GSH-Px activities of the ARPE-19 and ACBRI 181 cells were determined spectrophotometrically at 412 nm and 37 °C. [42]. The protein content of the cells was determined according to the method described by Lowry et al. [43].

### 4.8. Measurement of Mitochondrial Membrane Potential

Determination of mitochondrial membrane depolarization was carried out by loading cells with 1 µM JC-1 and measuring the resulting fluorescence intensity in an automatic microplate reader (Tecan Infinite M200 for ARPE-19 cells and SpectroStar Nano [BMC LabTech] for ACBRI 181 cells), as previously described [18]. The fluorescence signal of the green JC-1 was assessed at an excitation wavelength of 485 nm and an emission wavelength of 535 nm, while the red signal was measured at an excitation wavelength of 540 nm and an emission wavelength of 590 nm. Data were presented as the fold increase over the control level (untreated samples).

### 4.9. Apoptosis Assay

Determination of apoptosis assay was performed by using a commercial kit, namely APOPercentage TM assay (Belfast, Nothern Ireland) [18]. The described assay is a dye-uptake assay that specifically stains apoptotic cells with a red dye. When the membrane of an apoptotic cell loses its asymmetry, the APOPercentage dye is actively transported into the cells, resulting in the staining of apoptotic cells. This process enables the detection of apoptosis using a spectrophotometer. Data were presented as the fold increase over the control level (untreated samples).

### 4.10. Assay for Determination of Caspase Activities

The cells were subjected to sonication, and the resulting cell lysates were then mixed with 2 mL of substrate solution (composed of 20 mm HEPES [pH 7.4], 2 mm EDTA, 0.1% CHAPS detergent, 5 mm DTT, and 8.25 µM of caspase substrate). The mixture was incubated for 1 h at 37 °C, following the previously described method [18]. The activities of caspase-3 and -9 were assessed by measuring the cleavage of their respective specific fluorogenic substrates (Ac-DEVD-AMC for caspase-3 and AC-LEHD-AMC for caspase-9). The substrate cleavage was measured using a fluorescence spectrophotometer with an excitation wavelength of 360 nm and an emission wavelength of 460 nm. Initial experiments confirmed that the presence of caspase-3 inhibitor DEVD-CMK or caspase-9 inhibitor z-LEHD-FMK prevented substrate cleavage for their respective caspases. The resulting data was calculated as fluorescence units per milligram of protein using Tecan Infinite M200 for ARPE-19 cells and SpectroStar Nano [BMC LabTech] for ACBRI 181 cells. Data were presented as the fold increase over the control level (untreated samples).

### 4.11. Measurement of VEGF Levels

For each well, 3 × 10^4^ ARPE-19 and ACBRI 181 cells were plated in 200 µL of cell culture medium on 48-well plates with a target confluency of 90%. The cells were incubated in the presence or absence of D-glucose or Se, and the cultivation medium was refreshed. After the designated incubation period at 37 °C in a 5% CO_2_ environment, the culture media were collected and assessed for VEGF levels using an ELISA kit (Sigma Aldrich, St. Louis, MO, USA), following the manufacturer’s protocol. The curved line analysis resulted in an R^2^ value of 0.999.

### 4.12. Statistical Analysis

Statistical significance was analyzed using the SPSS program (SPSS, Chicago, IL, USA). To compare the effects of different treatments, statistical significance was determined by using Kruskal–Wallis test, Mann–Whitney *U*-tests, and post hoc tests. The *p*-value < 0.05 showed a statistically significant difference. Data were expressed as means ± SD of the number of determinations.

## Figures and Tables

**Table 1:** Effects of Se and different dosages of D-glucose on glutathione peroxidase (GSH-Px) activity, reduced glutathione (GSH), lipid peroxidation (LP), and vascular endothelial growth factor (VEGF) levels of ARPE-19.

Parameters/Groups	Control	Se	5 mM	5 mM + Se	20 mM	20 mM + Se	40 mM	40 mM + Se	80 mM	80 mM + Se
**LP** (µMol/g protein)	13.26 ± 1.24	10.76 ± 1.34 ^a^	15.89 ± 1.23 ^a,b^	13.87 ± 1.38 ^b,c^	25.93 ± 2.35 ^a,b,c,d^	20.16 ± 2.41 ^a,b,c,d,e^	26.18 ± 1.76 ^a,b,c,d,e,f^	24.22 ± 2.43 ^a,b,c,d,e,f,g^	28.53 ± 2.47 ^a,b,c,d,e,f,g,h^	27.11 ± 2.46 ^a,b,c,d,e,f,g,h,i^
**GSH** (µMol/g protein)	6.16 ± 0.44	14.12 ± 0.44 ^a^	5.48 ± 1.41 ^a,b^	7.47 ± 1.64 ^a,b,c^	4.33 ± 1.26 ^a,b,c,d^	5.78 ± 1.36 ^a,b,d,e^	4.18 ± 1.27 ^a,b,c,d,f^	5.92 ± 1.24 ^a,b,d,e,g^	3.78 ± 0.64 ^a,b,c,d,e,f,g,h^	5.17 ± 1.57 ^a,b,d,e,g,i^
**GSH-Px** (IU/g protein)	15.62 ± 2.14	19.43 ± 2.34 ^a^	14.42 ± 2.32 ^b^	16.01 ± 2.78 ^b,c^	18.65 ± 1.84 ^a,c,d^	17.71 ± 2.36 ^a,b,c,d^	20.82 ± 2.36 ^a,c,d,e,f^	18.76 ± 1.77 ^a,c,d,f^	22.52 ± 2.18 ^a,b,c,d,e,f,h^	20.43 ± 4.41 ^a,c,d,e,f,h,i^
**VEGF** (pg/mL)	41.18 ± 2.48	33.25 ± 3.21 ^a^	54.17 ± 5.16 ^a,b^	50.16 ± 5.24 ^a,b,c^	63.45 ± 4.28 ^a,b,c,d^	57.13 ± 3.47 ^a,b,c,d,e^	67.28 ± 5.18 ^a,b,c,d,e,f^	58.14 ± 4.27 ^a,b,c,d,e,g^	69.38 ± 4.37 ^a,b,c,d,e,f,h^	61.21 ± 3.18 ^a,b,c,d,e,f,g,h,i^

*n* = 8, mean ± SD. ^a^ *p* < 0.001 vs. control, ^b^ *p* < 0.05 vs. Se, ^c^ *p* < 0.001 vs. 5 mM, ^d^ *p* < 0.001 vs. 5 mM + Se, ^e^ *p* < 0.001 vs. 20 mM, ^f^ *p* < 0.001 vs. 20 mM + Se, ^g^ *p* < 0.001 vs. 40 mM, ^h^ *p* < 0.001 vs. 40 mM + Se, ^i^ *p* < 0.001 vs. 80 mM.

**Table 2:** Effects of Se and different dosages of D-glucose on glutathione peroxidase (GSH-Px) activity, reduced glutathione (GSH), lipid peroxidation (LP), and vascular endothelial growth factor (VEGF) levels of primary human retinal microvascular endothelial cells (ACBRI 181).

Parameters/Groups	Control	Se	5 mM	5 mM + Se	20 mM	20 mM + Se	40 mM	40 mM + Se	80 mM	80 mM + Se
**LP** (µMol/g protein)	14.37 ± 1.19	9.81 ± 1.28 ^a^	16.74 ± 1.14 ^a,b^	14.87 ± 1.38 ^b,c^	27.41 ± 1.21 ^a,b,c,d^	21.13 ± 1.85 ^a,b,c,d,e^	28.41 ± 1.24 ^a,b,c,d,e,f^	25.28 ± 2.14 ^a,b,c,d,e,f,g^	27.13 ± 2.47 ^a,b,c,d,e,f,g,h^	29.14 ± 2.13 ^a,b,c,d,e,f,g,h,i^
**GSH** (µMol/g protein)	5.18 ± 0.38	13.23 ± 0.52 ^a^	5.52 ± 1.24 ^a,b^	7.56 ± 1.28 ^a,b,c^	5.13 ± 1.17 ^a,b,c,d^	5.59 ± 1.24 ^a,b,d,e^	4.23 ± 1,34 ^a,b,c,d,f^	6.13 ± 1.41 ^a,b,d,e,g^	3.41 ± 0.27 ^a,b,c,d,e,f,g,h^	5.39 ± 1.21 ^a,b,d,e,g,i^
**GSH-Px** (IU/g protein)	14.27 ± 2.11	20.37 ± 1.87 ^a^	14.93 ± 2.15 ^b^	17.13 ± 2.14 ^b,c^	10.48 ± 1.23 ^a,c,d^	17.51 ± 2.36 ^a,b,c,d^	11.67 ± 2.56 ^a,c,d,e,f^	17.83 ± 1.41 ^a,c,d,f^	12.41 ± 2.07 ^a,b,c,d,e,f,h^	20.43 ± 2.12 ^a,c,d,e,f,h,i^
**VEGF** (pg/mL)	39.22 ± 2.13	31.17 ± 2.17 ^a^	52.29 ± 4.24 ^a,b^	48.13 ± 4.16 ^a,b,c^	60.17 ± 3.17 ^a,b,c,d^	55.17 ± 4.41 ^a,b,c,d,e^	65.17 ± 4.24 ^a,b,c,d,e,f^	55.43 ± 4.19 ^a,b,c,d,e,g^	67.51 ± 3.81 ^a,b,c,d,e,f,h^	57.21 ± 4.15 ^a,b,c,d,e,f,g,h,i^

*n* = 8, mean ± SD. ^a^ *p* < 0.001 vs. control, ^b^ *p* < 0.05 vs. Se, ^c^ *p* < 0.001 vs. 5 mM, ^d^ *p* < 0.001 vs. 5 mM + Se, ^e^ *p* < 0.001 vs. 20 mM, ^f^ *p* < 0.001 vs. 20 mM + Se, ^g^ *p* < 0.001 vs. 40 mM, ^h^ *p* < 0.001 vs. 40 mM + Se, ^i^ *p* < 0.001 80 mM.

## Data Availability

The data are contained within this article.

## Sample Availability

Samples of the compounds are available from the authors.
